# Heterogeneity of beta-cell function in subjects with multiple islet autoantibodies in the TEDDY family prevention study - TEFA

**DOI:** 10.1186/s40842-021-00135-6

**Published:** 2022-01-05

**Authors:** Maria Månsson Martinez, Lampros Spiliopoulos, Falastin Salami, Daniel Agardh, Jorma Toppari, Åke Lernmark, Jukka Kero, Riitta Veijola, Päivi Tossavainen, Sauli Palmu, Markus Lundgren, Henrik Borg, Anastasia Katsarou, Helena Elding Larsson, Mikael Knip, Marlena Maziarz, Carina Törn, and the TEDDY-Family (TEFA) Study Group, Anita Ramelius, Ida Jönsson, Rasmus Bennet, Birgitta Sjöberg, Åsa Wimar, Jessica Melin, Maria Ask, Anne Wallin, Monika Hansen, Susanne Hyberg, Karin Ottosson, Jenny Bremer, Ulla-Marie Carlsson, Ulrika Ulvenhag, Anette Sjöberg, Marielle Lindström, Lina Fransson, Fredrik Johansen, Kobra Rahmati, Zeliha Mestan, Evelyn Tekum-Amboh, Silvija Jovic, Joanna Gerardsson, Emelie Ericson-Hallström, Sofie Åberg, Sara Sibthorpe, Elina Mäntymäki, Sini Vainionpää, Minna Romo, Zhian Othmani, Eeva Varjonen, Sanna Jokipuu, Satu Ruohonen, Laura Leppänen, Petra Rajala, Eija Riski, Miia Kähönen, Minna-Liisa Koivikko, Tea Joensuu, Heidi Alanen, Teija Mykkänen, Tiina Latva-aho, Minna-Liisa Koivikko, Aino Stenius, Paula Ollikainen, Marika Korpela, Katja Multasuo, Päivi Salmijärvi, Pieta Kemppainen, Merja Runtti, Riitta Päkkilä, Irene Viinikangas, Sinikka Pietikäinen, Tuula Arkkola

**Affiliations:** 1 Department of Clinical Sciences, Lund University CRC, Skåne University Hospital, Jan Waldenströms gata 35, Box 503 32, SE-214 28 Malmö, Sweden; 2 Department of Pediatrics, Turku University Hospital, Turku, Finland; 3 Research Centre for Integrative Physiology and Pharmacology, Institute of Biomedicine, and Centre for Population Health Research, University of Turku, Turku, Finland; 4 Department of Pediatrics, PEDEGO Research Unit, MRC Oulu, University of Oulu, Oulu, Finland; 5 Department of Children and Adolescents, Oulu University Hospital, Oulu, Finland; 6 Department of Pediatrics, Tampere Center for Child, Adolescent and Maternal Health Research, Tampere University Hospital, Tampere, Finland; 7 Research Program for Clinical and Molecular Metabolism, Faculty of Medicine, University of Helsinki, Helsinki, Finland

**Keywords:** Islet autoantibodies, beta-cell function, Glucose metabolism, Continuous glucose monitoring

## Abstract

**Background:**

Individuals with multiple islet autoantibodies are at increased risk for clinical type 1 diabetes and may proceed gradually from stage to stage complicating the recruitment to secondary prevention studies. We evaluated multiple islet autoantibody positive subjects before randomisation for a clinical trial 1 month apart for beta-cell function, glucose metabolism and continuous glucose monitoring (CGM). We hypothesized that the number and type of islet autoantibodies in combination with different measures of glucose metabolism including fasting glucose, HbA1c, oral glucose tolerance test (OGTT), intra venous glucose tolerance test (IvGTT) and CGM allows for more precise staging of autoimmune type 1 diabetes than the number of islet autoantibodies alone.

**Methods:**

Subjects (*n* = 57) at 2–50 years of age, positive for two or more islet autoantibodies were assessed by fasting plasma insulin, glucose, HbA1c as well as First Phase Insulin Response (FPIR) in IvGTT, followed 1 month later by OGTT, and 1 week of CGM (*n* = 24).

**Results:**

Autoantibodies against GAD65 (GADA; *n* = 52), ZnT8 (ZnT8A; *n* = 40), IA-2 (IA-2A; *n* = 38) and insulin (IAA; *n* = 28) were present in 9 different combinations of 2–4 autoantibodies. Fasting glucose and HbA1c did not differ between the two visits. The estimate of the linear relationship between log2-transformed FPIR as the outcome and log2-transformed area under the OGTT glucose curve (AUC) as the predictor, adjusting for age and sex was − 1.88 (− 2.71, − 1.05) *p* = 3.49 × 10–5. The direction of the estimates for all glucose metabolism measures was positive except for FPIR, which was negative. FPIR was associated with higher blood glucose. Both the median and the spread of the CGM glucose data were significantly associated with higher glucose values based on OGTT, higher HbA1c, and lower FPIR. There was no association between glucose metabolism, autoantibody number and type except that there was an indication that the presence of at least one of ZnT8(Q/R/W) A was associated with a lower log2-transformed FPIR (− 0.80 (− 1.58, − 0.02), *p* = 0.046).

**Conclusions:**

The sole use of two or more islet autoantibodies as inclusion criterion for Stage 1 diabetes in prevention trials is unsatisfactory. Staging type 1 diabetes needs to take the heterogeneity in beta-cell function and glucose metabolism into account.

**Trial registration:**

ClinicalTrials.gov identifier: NCT02605148, November 16, 2015

**Supplementary Information:**

The online version contains supplementary material available at 10.1186/s40842-021-00135-6.

## Background

The clinical onset of autoimmune type 1 diabetes is preceded by a prodrome of immune-associated dysfunction of the pancreatic islet beta-cells resulting in an eventual failure to produce sufficient amounts of insulin to maintain normal blood glucose. The aetiology of the disease is thought to include environmental factors such as virus [1, 2] that would trigger islet autoimmunity marked by the appearance of either autoantibodies against insulin (IAA) or GAD65 (GADA) as the first appearing autoantibody [3–5]. In about 60% of children who developed either IAA first or GADA first, a second autoantibody appeared within 1 year [6]. Children, as well as adults, with two or more islet autoantibodies proceed to develop diabetes but it may take up to 20 years before the clinical onset [7]. Hence, the sub-clinical autoimmune process resulting in the destruction and dysfunction of beta-cells begins months (in very young children) or years before the appearance of the classical clinical symptoms of type 1 diabetes and is reflected in a decreased first phase insulin response (FPIR) [[8] related to the number of islet autoantibodies and genetic factors other than HLA [9, 10]]. At the onset of clinical symptoms, only a small fraction of the functional beta-cell mass is thought to be left.

The gradual loss of pancreatic islet beta-cell function is associated with both a cellular and a marked humoral immune response reflected by autoantibodies against several beta-cell autoantigens. At the clinical onset, about 95% of the patients have one or several autoantibodies against islet antigen-2 (IA-2A) and ZnT8 transporter (ZnT8A) in addition to GADA and IAA [11, 12]. These autoantibodies not only predict the clinical onset of type 1 diabetes [7], but may also be biomarkers of the pathogenic process leading to clinical diagnosis. The autoantibody biomarkers are therefore used to screen subjects at increased genetic risk of type 1 diabetes, such as first-degree relatives [13]. In a previous analysis, we showed that single autoantibody positive subjects had normal beta-cell function. Compared to subjects with two autoantibodies, subjects positive for three or more autoantibodies had a lower FPIR [8, 14]. This observation supports the view that the beta-cell function had deteriorated when the subjects progressed from positivity for two to having three autoantibodies. It is also consistent with the observation that children 4–9 years of age with GADA and at least one more autoantibody, had variable glucose metabolism [15].

In the present work, we wanted to test the hypothesis that the number and type of islet autoantibodies in combination with five different measures of glucose metabolism including fasting glucose, HbA1c, oral glucose tolerance test (OGTT), intra venous glucose tolerance test (IvGTT) and continuous glucose monitoring (CGM) allows for more precise staging of autoimmune type 1 diabetes than the number of islet autoantibodies alone. Specifically, the aim was to determine whether in subjects positive for at least two islet autoantibodies and to be randomized in a clinical intervention trial NCT02605148. (1) the number and (2) the types of autoantibodies were associated with any of the baseline measures of beta-cell function and glucose metabolism.

## Methods

### Study population

The study included 57 research subjects who agreed to participate in a first visit with an IvGTT and 1 month later in a second visit with an OGTT. The subjects were relatives to research subjects who participated in longitudinal studies because they were born with increased genetic risk for type 1 diabetes including the TEDDY study [16, 17]. There were 15/57 (26%) who had a father (*n* = 6), mother (*n* = 3), sibling (*n* = 3) or a child (*n* = 4) with type 1 diabetes. The inclusion and exclusion criteria are summarized in Supplementary Table 1, Additional file.

The characteristics of the *n* = 57 subjects recruited from Sweden and Finland are summarized in Table 1. The median (interquartile range (IQR)) age was 11.0 years (8.0, 16.0), the country-specific median (IQR) age was 13.0 years (10.0, 16.0) for subjects in Sweden and 8.0 years (3.3, 13.0) for subjects in Finland. Of the 57 subjects, 30 (52.6%) were female, and all subjects were within the normal range of height, weight and HbA1c (Table 1).

**Table 1:** Characteristics of subjects enrolled in the TEFA study (*n* = 57)

Characteristics	***n*** = 57
Swedish participants (n, %)	35 (61.4%)
Finnish participants (n, %)	22 (38.6%)
Age (years) (median, IQR)	11.3 (8.4, 16.0)
Females (n, %)	30 (52.6%)
First-degree relatives (vs. general population) (n, %)	17 (29.8%)
Weight (kg) (median, IQR)	44.8 (25.9, 60.3)
Height (cm) (median, IQR)	151.0 (133.9, 166.4)
HbA1c (mmol/mol) (median, IQR)	33.0 (31.0, 36.0)
HOMA2%B (median, IQR)	91.9 (65.7, 120.6)
HOMA2%S (median, IQR)	87.9 (66.2, 120.8)

*IQR* Interquartile range

In addition to demographic information, we have data on autoantibodies at both visit 1 and 2, fasting glucose, HbA1c, IvGTT (visit 1), OGTT (visit 2), and CGM for 24/57 subjects for 1 week after visit 2. Thus, for most analyses the sample size was *n* = 57, and for analyses involving CGM data the sample size was *n* = 24.

### Blood samples and analyses

Blood samples were analyzed for plasma glucose, serum insulin and C-peptide, HbA1c and autoantibodies. The analyses were performed at local clinical chemistry laboratories as described earlier [14]. In an attempt to harmonize assays for serum insulin, samples were in addition analyzed by using ELISA in the Diabetes Research Laboratory, Oulu, Finland. The serum insulin assays in Oulu and in Malmö were correlated (r^2^ = 0.985) and all measurements in Oulu were normalized to Malmö levels in mIE/L.

Swedish samples for GADA, IA-2A, IAA and ZnT8A were analyzed in Malmö, Sweden and in Oulu, Finland for the Finnish samples except ZnT8(W,R) A which were analyzed in Helsinki, Finland. All three laboratories participate in the Islet Autoantibody Standardization Program (IASP) [18]. The GADA assay was 64% sensitive and 94% specific for the samples analyzed in Malmö and 60% sensitive and 97.8% specific for those analyzed in the DIPP laboratory in Oulu. The IA-2A assay was 62% sensitive and 100% specific for the samples analyzed in Malmö and 76% sensitive and 100% specific in Oulu. The IAA assay was 18% sensitive and 96.7% specific for the samples analyzed in Malmö and 40% sensitive and 96.7% specific in Oulu. ZnT8(R/W/Q)A (three variants at position 325) were analyzed in Malmö while the laboratory in Helsinki determined ZnT8A simultaneously for both the ZnT8R and W variants. The IASP results were 66% sensitive and 100% specific for ZnT8(RWQ)A in Malmö and 74% sensitive and 100% specific for ZnT8(R,W)A in Helsinki.

### Glucose metabolism measures

Fasting and stimulated C-peptide and insulin levels were measured at both visits 1 and 2. HbA1c was analyzed using a spectrophotometric assay (Capillary 3 Tera; Sebia, Paris, France). The reference values for samples analyzed for HbA1c were 27–42 mmol/mol in Malmö and 20–42 mmol/L in Oulu and Turku. The reference values in Malmö were in subjects 1 months – 18 years of age: p-glucose 1 month – 18 yrs.: 3.3–5.6 mmol/L and above 18 yrs.: 4.2–6.3 mmol/L, s-insulin < 25 mIE/ L mIE/L, s-C-peptide: 0.37–1.5 nmol/ L. The corresponding reference values were in Oulu: p-glucose 4.2–6.0 mmol/L, s-insulin 5–20 mU/L mIE/L, s-C-peptide: > 0.9 nmol/ L, and in Turku: p-glucose 4–6 mmol/L, s-insulin 2.6–25 mU/L mIE/L, s-C-peptide 0.37–1.47 nmol/L. All sample reference ranges apply to fasting condition.

### Statistical methods

The autoantibody data was coded as a binary variable for each autoantibody (GADA, IAA, IA-2A, ZnT8(W/R/Q)A). We also considered the number of autoantibodies, where GADA, IAA and IA-2A each counted as 1 and any of ZnT8(W/R/Q)A counted as 1, thus the autoantibody count ranged from 2 to 4 for all subjects. The autoantibody status combinations were grouped into 4 categories, based on three most common combinations forming a category each, and the remaining combinations forming the fourth group. Fasting glucose and HbA1c values were evaluated using non-parametric paired Wilcoxons rank test to compare the distributions of fasting glucose and HbA1c between visits 1 and 2. The glucose values from an OGTT were used as individual values at a given time point (− 10 to 120 min), as well as summarized as the area under the OGTT curve (AUC). The FPIR values estimated from IvGTT were right-skewed and were log2-transformed. We performed a descriptive analysis evaluating the integrity of the data and visualizing the univariate and bivariate relationships between the different measures of glucose, as well as between glucose and autoantibody count and type, as well as FPIR and HbA1c.

Linear regression, adjusting for age and sex, was used to evaluate whether there was an association between each of 10 glucose metabolism measures (as the outcome) and autoantibody (1) status, (2) count, or (3) type (as predictors). The ten measures of glucose metabolism we considered were (A) OGTT 2 h glucose, (B) log2-transformed OGTT glucose AUC, (C) log2-transformed OGTT C-peptide AUC, (D) log2-transformed OGTT insulin AUC, (E) the median glucose value based on a CGM from a 7-day sampling every 5 min, or (F) the difference between the 75th and 25th percentiles of glucose values based on a CGM from a 7-day sampling every 5 min, (G) HbA1c, (H) log2-transformed FPIR, as well as two measures of homeostasis model assessment (HOMA): (I) log2-transformed HOMA2-%B quantifying beta cell function, and (J) log2-transformed HOMA2-%S quantifying insulin sensitivity. HOMA2-%B and HOMA2-%S were calculated using the HOMA2 model available from: www.dtu.ox.ac.uk/homacalculator/. We used the “Insulin” tab in the “Excel spreadsheet implementation” with fasting glucose (in mmol/L) and fasting insulin measured using radioimmunoassay (in pmol/L) as inputs [19, 20].

Simple linear regression was used to evaluate whether there is an association between the different glucose metabolism measures. First, we estimated the association between log2-transformed glucose AUC and log2-transformed FPIR. We then estimated the associations between the 7-day CGM glucose measurements summarized in two ways and treated as the outcome ((A) the median value for each individual or (B) the difference between 75th and 25th percentiles of the 7-day CGM glucose values), and one of four glucose metabolism measures treated as a predictor (log2-tranformed FPIR, HbA1c, 2 h OGTT glucose, or log2-transformed OGTT glucose AUC), adjusting for age and sex. All analyses were performed in R (www.r-project.org).

## Results

**Fig. 1:**
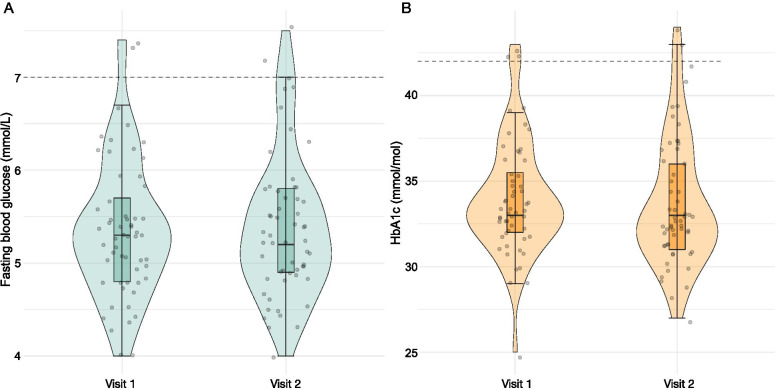
The distribution of fasting glucose (mmol/L) (1A) and HbA1c (mmol/mol) (1B) at visits 1 and 2 (*n* = 57). The boxplot indicates the median, the interquartile range, the violin around the boxplot shows the shape of the distribution of values at each visit, with individual data points shown in grey. The fasting glucose was measured 10 min before the start of IvGTT at visit 1 and OGTT at visit 2. The dashed lines at 7 mmol/L in 1A and at 42 mmol/mol in 1B indicate the World Health Organization thresholds above which subject is considered to be diabetic. Fasting glucose measurements were not found to differ between visits 1 and 2, nor did HbA1c at visits 1 and 2 (Wilcoxon test p-value 0.99 and 0.27, respectively)

There were nine autoantibody combinations identified among the 57 subjects (Table 2). The three most frequent autoantibody combinations were: (A) IAA, GADA, IA-2A and at least one of ZnT8(W/Q/R)A (*n* = 12; 21.1%), (B) GADA, IA-2A and at least one of ZnT8(W/Q/R)A (*n* = 10; 17.5%) as well as (C) GADA and at least one of ZnT8(W/Q/R)A (*n* = 10; 17.5%) (Table 2). The distribution of the fasting glucose measurements at visit 1 and 2 (Fig. 1A), as well as HbA1c at visit 1 and 2 (Fig. 1B) show that the fasting glucose and HbA1c, remained stable between the two visits (*p*-value 0.99, 0.27, respectively).

**Table 2:** Autoantibody status combinations detected for each subject up to visit 2 (*n* = 57)

IAA (n positive subjects = 28)	GADA (n positive subjects = 52)	IA-2A (n positive subjects = 38)	ZnT8W/Q/R (n positive subjects = 40)	Autoantibody count	n (%) subjects with a given autoantibody combination	Autoantibody combination group
+	+	+	+	4	12 (21.1)	A
–	+	+	+	3	10 (17.5)	B
–	+	–	+	2	10 (17.5)	C
+	+	+	–	3	4 (7.0)	D
+	+	–	+	3	3 (5.3)	D
+	+	–	–	2	6 (10.5)	D
+	–	+	+	3	3 (5.3)	D
–	+	+	–	2	7 (12.3)	D
–	–	+	+	2	2 (3.5)	D

The autoantibody combination group in the right-most column was defined based on the three most frequent autoantibody combinations, forming groups A-C (*n* = 12, 10, 10, respectively), with group D (*n* = 25) comprised of all remaining autoantibody combinations

**Fig. 2:**
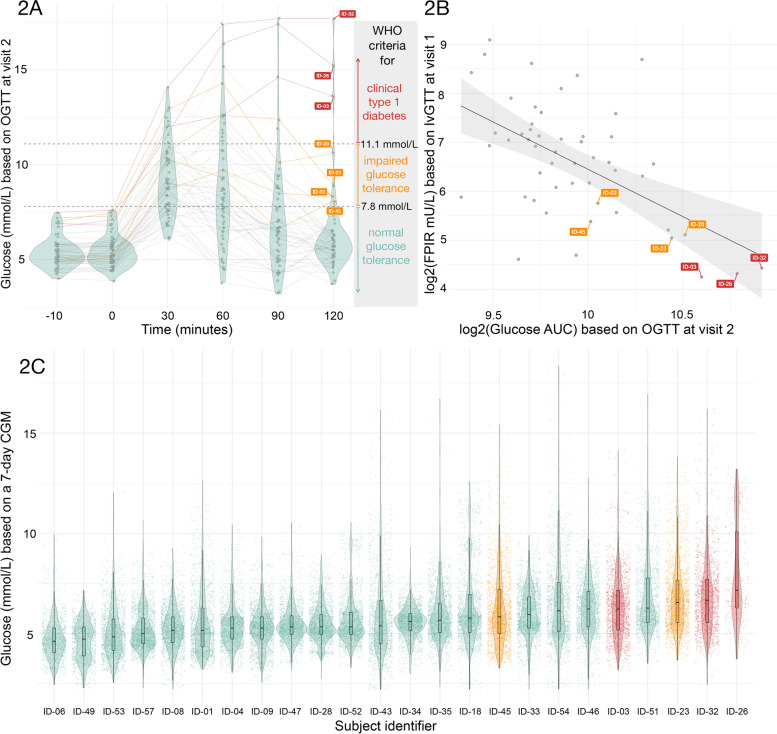
Assessment of glucose metabolism based on OGTT (2A and B), IvGTT (2B) (*n* = 57) and CGM (2C) (*n* = 24). In panel 2A we show the individual trajectories of blood glucose measured using a 2-h OGTT test at visit 2. Based on glucose values at minute 120, we identified 7 subjects (labelled with red and orange subject labels) would be considered to have impaired glucose tolerance (orange labels) or to have clinical type 1 diabetes (red labels) according to the World Health Organization (see grey panel in 2A). In panel 2B we show a scatterplot and a regression line between the log2-transformed area under the curve (AUC) of OGTT glucose measurements versus the log2-transformed FPIR measurements from IvGTT at visit 1. The subjects labelled in orange and red correspond to those in panel 2A. In panel 2C we present the distributions of the glucose measurements obtained from the Continuous Glucose Monitor (CGM) over a 7-day period starting at visit 2. The subjects were sorted according to an increasing median glucose value. The individual glucose measurements are shown as points, with the boxplots showing the median and interquartile range, and the violin plot showing the distribution of the CGM glucose values for a given individual. The subjects shown in orange and red correspond to those in panels 2A and 2B

The relationships between three different measures of glucose metabolism are illustrated in Fig. 2: glucose based on OGTT (panels 2A and 2B), FPIR based on IvGTT (panel 2B), and glucose based on a 7-day CGM (panel 2C). We noted three subjects (shown in red) who at 120 min remain above the ADA (American Diabetes Association) threshold for diabetes (11.1 mmol/L), as well as four subjects with impaired glucose tolerance (shown in orange) according to the ADA guidelines (Fig. 2A). Those seven subjects tended to have lower FPIR values and higher log2-transformed OGTT glucose AUC compared to the other subjects. The estimate of the linear relationship between log2-transformed FPIR as the outcome and log2-transformed OGTT glucose AUC as the predictor, adjusting for age and sex was − 1.88 (− 2.71, − 1.05) *p* = 3.49 × 10^− 5^ (Supplementary Table 2, Additional file)*.* Higher measures of log2-transformed OGTT glucose AUC were associated with lower log2-transformed FPIR mU/L, specifically, the doubling in the OGTT glucose AUC value was associated with a FPIR (mU/L) value 6.6 (2.9, 15.0) times lower (Fig. 2B).

The 7-day CGM glucose data for 24 subjects for whom the CGM data was available are shown in Fig. 2C. The CGM profiles are summarized using boxplots overlayed with violin plots showing the range and the distribution of the glucose values for each individual over the course of 7 days. The spread of the data, shown both as a boxplot and a density around it, seemed to increase as the median CGM glucose value increased for subjects shown from left to right, from lowest to highest median CGM glucose value. The individual CGM data shown in red corresponds to the three individuals in red in panels 2A and 2B. Two of the four subjects with impaired glucose tolerance are shown in orange (the CGM data for the other two individuals in that group was not available).

**Fig. 3:**
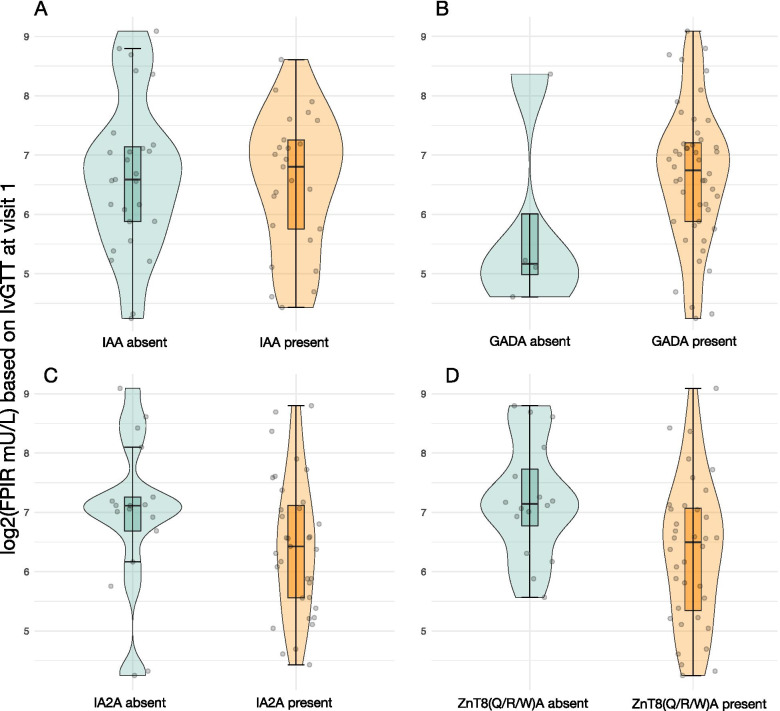
FPIR (mU/L) levels and autoantibody status. The distribution of log2-transformed first-phase insulin response (FPIR) (mU/L) stratified by presence of IAA, GADA, IA-2A or ZnT8(Q/R/Q)A (*n* = 57). The boxplot indicates the median, the interquartile range, the violin around the boxplot shows the shape of the distribution, with individual data points shown in grey. Based on the model in Table 3 with the status of the four antibodies as the main predictor, adjusted for age and sex, log2(FPIR) was not found to be statistically significantly different depending on the status of IAA, GADA or IA-2A (*p* = 0.564, 0.637, 0.262), but there was some evidence suggesting that FPIR is lower for those with at least one of ZnT8(Q/R/W)A present compared to those with no ZnT8(Q/R/W)A (1.74 mU/L lower (95% CI = 1.01, 2.99), *p* = 0.046)

The results of the analysis to evaluate the association between six glucose metabolism measures (from OGTT, IvGTT and CGM) and autoantibody count, status and type, are summarized in Supplementary Table 3, Additional file. There were no associations found between glucose metabolism and islet autoantibodies. There seemed to be an indication that the presence of at least one of ZnT8(Q/R/W)A was associated with a lower log2-transformed FPIR mU/L (− 0.80 (− 1.58, − 0.02), *p* = 0.046), but we consider this as a hypothesis-generating result, due to the overall number of tests performed in this analysis (Table 3, Fig. 3).

**Table 3:** The estimates and the 95% confidence intervals of the association between log2-transformed FPIR (mU/L) and autoantibody status, type or count, adjusted for age and sex

Covariates	Model 1 (***n*** = 52)	Model 2 (***n*** = 52)	Model 3 (***n*** = 52)
	Est (95% CI) ***p***-value	Est (95% CI) ***p***-value	Est (95% CI) ***p***-value
Autoantibody status (positive vs. negative)			
IAA	−0.20 (− 0.89, 0.49) 0.564		
GADA	0.32 (− 1.02, 1.65) 0.637		
IA-2A	−0.40 (− 1.12, 0.31) 0.262		
ZnT8(W/Q/R)A	**−0.80 (−1.58, − 0.02) 0.046**		
Number of autoantibodies		−0.39 (− 0.83, 0.05) 0.084	
Autoantibody combination group:			
A vs. D			−0.45 (−1.39, 0.49) 0.345
B vs. D			−0.68 (−1.64, 0.27) 0.158
C vs. D			−0.17 (−1.18, 0.84) 0.736
Age (per 10 years)	0.15 (−0.16, 0.45) 0.343	0.09 (− 0.23, .040) 0.580	0.17 (− 0.16, 0.49) 0.307
Male vs. female	− 0.14 (− 0.83, 0.56) 0.695	−0.08 (− 0.82, 0.65) 0.818	−0.24 (− 0.98, 0.51) 0.525

The estimates and the 95% confidence intervals of the association between log2-transformed FPIR (mU/L) as the outcome and three measures of autoantibody status as predictors (status, count, combination group), adjusting for age and sex, estimated using linear models. The autoantibody information used as the main predictors was modeled as: the autoantibody status for IAA, GADA, IA-2A and any of ZnT8(W/Q/R) A, with negative status being the reference, the number of autoantibodies detected (possible values were 2, 3, or 4), and the autoantibody status combination group A-D (see Table 2) with group D as the reference

We noted that ZnT8(Q/R/W)A was the only autoantibody measure that, in our analysis, indicates a consistent trend with all glucose metabolism measures. Specifically, the direction of the estimates for all glucose metabolism measures was positive with higher values of glucose AUC and negative for FPIR with lower first phase insulin response. Additionally, we noted an association between log2-transformed OGTT C-peptide AUC, as well as log2-transformed OGTT insulin AUC and age (Supplementary Table 3, Additional file).

The association between the two summaries of the 7-day CGM glucose measurements and glucose metabolism measures based on OGTT and IvGTT are summarized in Supplementary Table 4 and Supplementary Fig. 1, Additional file. The results are statistically significant for all associations tested, with both the median and the spread of the CGM glucose data being associated with higher glucose values based on OGTT, higher HbA1c, and lower FPIR (Supplementary Table 4 and Supplementary Fig. 1, Additional file).

## Discussion

The present study provides novel insights into the heterogeneity of islet autoantibody positive subjects in Stage 1 or Stage 2 of autoimmune type 1 diabetes. By combining fasting plasma glucose at two different visits 1 month apart with HbA1c, IvGTT for FPIR at the first visit and OGTT at the second visit followed by a 7-day CGM, it was possible to delineate the research subjects into three categories. First, the major group of two or more autoantibody positive subjects did not reveal signs of deteriorated glucose metabolism in any of the five measurements. This is of interest as research subjects belonging to this category varied from having two to four autoantibodies. Second, presenting with fasting glucose in the normal range, there were four (4/57) subjects (ID-02, ID-45, ID-23 and ID-20) who showed impaired glucose tolerance since the 120-min glucose values varied were within the 7.8–11.1 mmol/l range (Fig. 2). All four had reduced FPIR as well correlating to increased log2-transformed glucose AUC (Fig. 2B). Third, three (3/57) subjects (ID-03, ID-26 and ID-32) who by 120 min OGTT showed diabetes values which correlated to reduced FPIR (Fig. 2B). None of these three subjects showed any symptoms of diabetes. One OGTT with diabetes values with no concurrent symptoms is not sufficient for a diabetes diagnosis according to the ADA recommendations [21]. A second OGTT was not carried out. Taken together, the research subjects represented individuals who were screened because they were relatives to a subject at increased genetic risk for type 1 diabetes who were followed in the TEDDY [17] or DiPiS [22] studies. Only 7/57 (12%) were screened because they had a first degree relative with type 1 diabetes. We found that the majority (50/57) had glucose metabolism and beta-cell function within the normal range while 7/57 deviated from normal. The majority would therefore qualify in a secondary prevention trial where the risk for clinical onset would be about 70% in 10 years [7]. The smaller group of seven Stage 2 individuals would on the other hand perhaps be more suitable to be recruited into a secondary prevention trial aiming at preserving a deteriorating beta-cell function such as the recent trial with teplizumab [23]. The major finding is therefore that staging subjects into Stage 1 or 2 are best performed by a baseline IvGTT (the short version for FPIR would suffice) followed by an OGTT prior to randomization into a Stage 1or Stage 2 trial, respectively. Alternatively, OGTT may suffice as FPIR did not add additional subjects with impaired glucose tolerance.

Although not all subjects participated with CGM, five out of these seven subjects did. As CGM correlated well to aberrant OGTT and FPIR, our data therefore suggest that CGM should be considered as one additional approach to stage multiple autoantibody positive subjects prior to being randomize in secondary prevention studies. The combination of glucose metabolism measures and information on autoantibody status, count and type is likely to provide a more accurate estimate on the stage in the autoimmune process leading to clinical onset of type 1 diabetes than autoantibody status alone. Using the autoantibody information alone to determine the stage of the disease process appears to result in an inaccurate staging of the disease.

The observation that the mere presence of ZnT8A (any of the three variants) seemed to be associated with a more advanced deteriorated beta-cell function merits further investigation. In a previous baseline study of 47 children, [24] we observed that number and levels of autoantibodies were not associated with glucose metabolism, except for an increased frequency and level of ZnT8QA in children with impaired glucose metabolism [15]. This preliminary confirmation is of particular interest as the possible association between ZnT8A and progression to clinical onset of diabetes in multiple autoantibody positive subjects is poorly understood. Newly diagnosed type 1 diabetes patients with the CC (ZnT8R/R) and CT (ZnT8R/W) genotypes of the rs13266634 SNP of the SLC30A8 gene had higher stimulated C-peptide levels the first year after onset compared with TT (ZnT8W/W) subjects [25, 26]. This is of interest as the SLC30A8 genotype may be important to consider as a factor that contributes to progression to clinical onset of diabetes. Patients with newly diagnosed type 1 diabetes who were positive for ZnT8A were more frequently of older age, had less ketoacidosis and carried more often HLA DQB1*06:04 [27, 28]. Screening of first-degree relatives using IA-2A and ZnT8A alone allowed identification of the majority of rapidly progressing siblings and offspring [29]. Although ZnT8A are only considered as a useful additional risk marker [30], perhaps considering their levels [31], our previous [15] and present observation would require further studies into the association between ZnT8(W/R/Q)A and deteriorating beta-cell function.

Our study has some limitations. The number of subjects with multiple islet autoantibodies was not large. The study group was also dominated by subjects who were relatives, not to type 1 diabetes patients, but rather to subjects identified at birth because they had an increased genetic risk for type 1 diabetes such as subjects from the DIPP [32], DiPiS [22] and TEDDY [16] studies. The inclusion criterion for these studies were merely increased genetic risk for type 1 diabetes rather than the proband had developed islet autoantibodies or type 1 diabetes. However, despite the small number of subjects, the findings on glucose metabolism were clearly statistically significant. Swedish subjects were older than Finnish subjects, but age was taken into account in statistical models, and there were no apparent differences in results between study sites.

The use of fasting glucose, IvGTT, or OGTT, alone or in combination, to randomize subjects with multiple islet autoantibodies into secondary prevention trials in Stage 1 or Stage 2 cohorts is much discussed. Alternative methods to consider may be assessment of the beta-cell mass by both acute insulin response to arginine at hyperglycemia (AIRmax), as a correlate of beta-cell mass, and beta-cell function by (IvGTT) [33]. The IvGTT and FPIR may detect an acceleration in the loss of beta-cell function which may not be seen by OGTT [34]. The baseline heterogeneity observed in earlier studies of multiple islet autoantibody positive subjects that complicates secondary prevention [35] needs further considerations perhaps by taking into account the endotypes of type 1 diabetes defined by whether IAA (primarily in HLA DR4-DQ8 subjects) or GADA (DR3-DQ2 subjects) would be considered as the first appearing islet autoantibody [3, 5, 32]. Apart from further studies on CGM, it will also be worthwhile to consider levels of the islet autoantibodies, in particular ZnT8A, other non-HLA genetic factors, measures of insulin sensitivity such as HOMA-IR [36] along with BMI and family history of type 2 diabetes.

## Conclusions

Our results indicate that research subjects to be randomized to clinical trials for prevention of type 1 diabetes can be categorized more accurately when OGTT, IvGTT, and CGM are used prior to inclusion into prevention studies in addition to measurement of islet autoantibodies.

## Supplementary Information

**Additional file 1.**

## Data Availability

Supplementary Tables 1–4 are available in Betacellfunction _ Spilipoulus _ el_al _ BMC _ additional_files (PDF). The datasets used and/or analysed during the current study, as well as the analysis code, are available from the corresponding author on reasonable request.

## Abbreviations

ADA: American Diabetes Association
CGM: Continuous Glucose Monitoring
DiPiS: Diabetes Prediction in Skåne
DIPP: Type 1 Diabetes Prediction and Prevention
FPIR: First Phase Insulin Response
HOMA: Homeostasis model assessment
GADA: GAD65 (glutamate decarboxylase 65) autoantibody
IAA: Insulin autoantibody
IA-2A: Islet antigen-2 autoantibody
IvGTT: Intravenous glucose tolerance test
OGTT: Oral glucose tolerance test
TEDDY: The Environmental Determinants of Diabetes in the Young
TrialNet: Type 1 Diabetes TrialNet
ZnT8(W/Q/R)A: Zinc transporter 8 autoantibody, any of the three variants at position 325
