# Supplementary material for: Heterogeneity of beta-cell function in subjects with multiple islet autoantibodies in the TEDDY family prevention study - TEFA
Source: Clin Diabetes Endocrinol. 2022 Jan 5;7:23. doi: 10.1186/s40842-021-00135-6 (PMC8728995; doi:10.1186/s40842-021-00135-6)
Supplement: Supplementary file 1 — Additional file 1. [file 40842_2021_135_MOESM1_ESM.pdf]

Additional file

with **Supplementary Tables 1-4** and **Supplementary Figure 1** accompanying the following manuscript:

Heterogeneity of beta-cell function in subjects with multiple islet autoantibodies in the TEDDY Family Prevention Study - TEFA

Maria Månsson Martinez, Lampros Spiliopoulos, Falastin Salami, Daniel Agard, Jorma Toppari, Åke Lernmark, Jukka Kero, Riitta Veijola, Päivi Tossavainen, Sauli Palmu, Markus Lundgren, Henrik Borg, Anastasia Katsarou, Helena Elding Larsson, Mikael Knip, Marlena Maziarz and Carina Törn and the TEDDY-Family (TEFA) Study Group

Version 2021.11.09

Supplementary Table 1. Inclusion and exclusion criteria for entry into the TEFA study.

**Inclusion criteria**
(1) Between two and 50 years of age
(2) At least two type 1 diabetes-associated autoantibodies measured at visit 0 or 1 in TEFA, or confirmed at two consecutive samples in other studies before inclusion in TEFA, OR, at least one type 1 diabetes-associated autoantibody (GADA, IAA, IA-2A or ZnT8R/W/QA) and impaired glucose metabolism as defined by WHO. (3) Written informed consent from research subject. If a child, also from the child's parents or legal acceptable representative(s) according to local regulations.

**Exclusion criteria**
(1) Ongoing treatment with immunosuppressant therapy (topical or inhaled steroids are accepted).
(2) Diabetes (either type 1 or 2).
(3) Treatment with any oral or injected anti-diabetic medications.
(4) Significantly abnormal hematology results at screening.
(5) Participation in other clinical trials with a new chemical entity within the previous 3 months.
(6) History of hypercalcemia.
(7) Presence of associated serious disease or condition.

Supplementary Table 2. The linear relationship between log2(FPIR) as the outcome and log2(OGTT glucose AUC) as the predictor, adjusting for age and sex.

Outcome	Covariates (predictors)	Model (n = 52) Est (95% CI) p-value
log2(FPIR)	log2(GLUCOSE_auc)	-1.88 (-2.71, -1.05) 0.0000349
	Age (per 10 years)	0.17 (-0.08, 0.43) 0.182
	Male vs. Female	-0.12 (-0.72, 0.48) 0.692

Supplementary Table 3 (part 1 of 4). The estimates and the 95% confidence intervals of the association between each combination of six measures of glucose metabolism (A-F), as well as G: HbA1c (mmol/mol), H: log2(FPIR mU/L), and two measures of homeostasis model assessment (I) log2-transformed HOMA2-%B quantifying beta cell function, and (J) log2-transformed HOMA2-%S quantifying insulin sensitivity as outcomes and three measures of autoantibody status as predictors (count, status, combination group), adjusting for age and sex, estimated using linear models. The six outcome measures of glucose metabolism are: A: OGTT 2hr glucose (mmol/L), B. log2(OGTT glucose AUC), C: HbA1c (mmol/mol), D. log2(FPIR ml/L)), E: the median glucose (mmol/L) value based on a CGM from a 7-day sampling every 5 minutes, and F: The difference between the 75th and 25th percentiles of glucose (mmol/L) values based on a CGM from a 7-day sampling every 5 minutes. The outcomes were log2-tranformed as needed. The autoantibody information used as the main predictors was modeled as: the number of autoantibodies (possible values were 2, 3, or 4), autoantibody status for IAA, GADA, IA-2A and any of ZnT8(W/Q/R)A, with negative status being the reference, and the autoantibody combination group A-D (see Table 2) with group D as the reference. (Panel H in blue was reported in the main manuscript as well, it is included here for completeness).

Outcome A: OGTT 2h glucose (mmol/L)

Covariates (predictors)	Model 1A (n = 57)<br>Est (95% CI) p-value	Model 2A (n = 57)<br>Est (95% CI) p-value	Model 3A (n = 57)<br>Est (95% CI) p-value
Number of autoantibodies	0.19 (-0.70, 1.07) 0.674		
IAA (positive vs. negative)		0.29 (-1.08, 1.67) 0.669	
GADA (positive vs. negative)		-0.07 (-2.60, 2.46) 0.955	
IA-2A (positive vs. negative)		-0.52 (-1.97, 0.94) 0.479	
ZnT8(W/Q/R)A (positive vs. negative)		1.10 (-0.48, 2.69) 0.169	
Autoantibody combination group:			
A vs. D			1.07 (-0.72, 2.87) 0.234
B vs. D			-0.07 (-1.96, 1.82) 0.941
C vs. D			-1.36 (-0.55, 3.26) 0.159
Age (per 10 years)	-0.34 (-0.96, 0.29) 0.287	-0.22 (-0.87, 0.43) 0.500	-0.24 (-0.86, 0.39) 0.446
Male vs. female	-0.93 (-2.32, 0.46) 0.186	-0.85 (-2.28, 0.58) 0.239	-0.89 (-2.30, 0.52) 0.211

Outcome B: log2(OGTT glucose AUC)

Covariates (predictors)	Model 1B (n = 57)<br>Est (95% CI) p-value	Model 2B (n = 57)<br>Est (95% CI) p-value	Model 3B (n = 57)<br>Est (95% CI) p-value
Number of autoantibodies	0.06 (-0.06, 0.18) 0.339		
IAA (positive vs. negative)		0.01 (-0.18, 0.21) 0.903	
GADA (positive vs. negative)		0.12 (-0.24, 0.47) 0.516	
IA-2A (positive vs. negative)		0.05 (-0.16, 0.25) 0.644	
ZnT8(W/Q/R)A (positive vs. negative)		0.14 (-0.08, 0.36) 0.216	
Autoantibody combination group:			
A vs. D			0.16 (-0.09, 0.41) 0.213
B vs. D			0.12 (-0.15, 0.39) 0.369
C vs. D			0.12 (-0.15, 0.39) 0.367
Age (per 10 years)	0.00 (-0.09, 0.09) 0.979	0.01 (-0.09, 0.10) 0.909	0.00 (-0.08, 0.09) 0.924
Male vs. female	0.03 (-0.16, 0.23) 0.737	0.05 (-0.15, 0.25) 0.624	0.05 (-0.15, 0.25) 0.636

Outcome C: log2(OGTT c-peptide AUC)

Covariates (predictors)	Model 1C (n = 56)<br>Est (95% CI) p-value	Model 2C (n = 56)<br>Est (95% CI) p-value	Model 3C (n = 56)<br>Est (95% CI) p-value
Number of autoantibodies	-0.05 (-0.32, 0.21) 0.678		
IAA (positive vs. negative)		0.00 (-0.41, 0.42) 0.983	
GADA (positive vs. negative)		0.31 (-0.44, 1.07) 0.407	
IA-2A (positive vs. negative)		-0.17 (-0.61, 0.28) 0.453	
ZnT8(W/Q/R)A (positive vs. negative)		-0.04 (-0.51, 0.43) 0.866	
Autoantibody combination group:			
A vs. D			0.18 (-0.36, 0.72) 0.496
B vs. D			-0.19 (-0.76, 0.38) 0.503
C vs. D			0.29 (-0.28, 0.87) 0.308
Age (per 10 years)	**0.32 (0.14, 0.51) 0.001**	**0.31 (0.12, 0.51) 0.002**	**0.36 (0.17, 0.55) <0.001**
Male vs. female	-0.16 (-0.58, 0.26) 0.442	0.03 (-0.16, 0.23) 0.744	-0.16 (-0.59, 0.26) 0.443

Supplementary Table 3 (part 2 of 4). The estimates and the 95% confidence intervals of the association between each combination of six measures of glucose metabolism (A-F), as well as G: HbA1c (mmol/mol), H: log2(FPIR mU/L), and two measures of homeostasis model assessment (I) log2-transformed HOMA2-%B quantifying beta cell function, and (J) log2-transformed HOMA2-%S quantifying insulin sensitivity as outcomes and three measures of autoantibody status as predictors (count, status, combination group), adjusting for age and sex, estimated using linear models. The six outcome measures of glucose metabolism are: A: OGTT 2hr glucose (mmol/L), B. log2(OGTT glucose AUC), C: HbA1c (mmol/mol), D. log2(FPIR ml/L)), E: the median glucose (mmol/L) value based on a CGM from a 7-day sampling every 5 minutes, and F: The difference between the 75th and 25th percentiles of glucose (mmol/L) values based on a CGM from a 7-day sampling every 5 minutes. The outcomes were log2-tranformed as needed. The autoantibody information used as the main predictors was modeled as: the number of autoantibodies (possible values were 2, 3, or 4), autoantibody status for IAA, GADA, IA-2A and any of ZnT8(W/Q/R)A, with negative status being the reference, and the autoantibody combination group A-D (see Table 2) with group D as the reference. (Panel H in blue was reported in the main manuscript as well, it is included here for completeness).

Outcome D: log2(OGTT insulin AUC)

Covariates (predictors)	Model 1D (n = 56)<br>Est (95% CI) p-value	Model 2D (n = 56)<br>Est (95% CI) p-value	Model 3D (n = 56)<br>Est (95% CI) p-value
Number of autoantibodies	-0.15 (-0.46, 0.15) 0.317		
IAA (positive vs. negative)		-0.20 (-0.69, 0.28) 0.406	
GADA (positive vs. negative)		0.07 (-0.81, 0.95) 0.869	
IA-2A (positive vs. negative)		0.00 (-0.51, 0.52) 0.997	
ZnT8(W/Q/R)A (positive vs. negative)		-0.34 (-0.90, 0.21) 0.218	
Autoantibody combination group:			
A vs. D			-0.11 (-0.75, 0.53) 0.727
B vs. D			-0.22 (-0.89, 0.46) 0.524
C vs. D			0.12 (-0.56, 0.80) 0.726
Age (per 10 years)	**0.25 (0.03, 0.46) 0.028**	0.22 (-0.01, 0.45) 0.061	**0.27 (0.04, 0.50) 0.022**
Male vs. female	-0.36 (-0.84, 0.13) 0.146	-0.35 (-0.85, 0.16) 0.174	-0.37 (-0.87, 0.14) 0.155

Outcome E: The median glucose (mmol/L) value based on a CGM from a 7-day sampling every 5 minutes

Covariates (predictors)	Model 1E (n = 24)<br>Est (95% CI) p-value	Model 2E (n = 24)<br>Est (95% CI) p-value	Model 3E (n = 24)<br>Est (95% CI) p-value
Number of autoantibodies	-0.10 (-0.44, 0.24) 0.564		
IAA (positive vs. negative)		0.11 (-0.64, 0.86) 0.762	
GADA (positive vs. negative)		0.01 (-1.26, 1.27) 0.992	
IA-2A (positive vs. negative)		-0.44 (-1.25, 0.37) 0.271	
ZnT8(W/Q/R)A (positive vs. negative)		0.22 (-1.05, 1.50) 0.718	
Autoantibody combination group:			
A vs. D			0.12 (-0.75, 0.98) 0.783
B vs. D			-0.26 (-1.29, 0.77) 0.602
C vs. D			0.38 (-0.63, 1.40) 0.437
Age (per 10 years)	0.21 (-0.67, 0.25) 0.357	-0.13 (-0.71, 0.45) 0.651	-0.21 (-0.67, 0.26) 0.361
Male vs. female	-0.26 (-0.87, 0.34) 0.370	-0.25 (-0.97, 0.47) 0.477	-0.28 (-0.95, 0.39) 0.397

Outcome F: The difference between the 75th and 25th percentiles of glucose (mmol/L) values based on a CGM from a 7-day sampling every 5 minutes

Covariates (predictors)	Model 1F (n = 24)<br>Est (95% CI) p-value	Model 2F (n = 24)<br>Est (95% CI) p-value	Model 3F (n = 24)<br>Est (95% CI) p-value
Number of autoantibodies	-0.21 (-0.53, 0.12) 0.201		
IAA (positive vs. negative)		-0.10 (-0.84, 0.63) 0.773	
GADA (positive vs. negative)		-0.32 (-1.56, 0.91) 0.587	
IA-2A (positive vs. negative)		-0.39 (-1.18, 0.40) 0.314	
ZnT8(W/Q/R)A (positive vs. negative)		0.15 (-1.09, 1.40) 0.798	
Autoantibody combination group:			
A vs. D			-0.18 (-1.02, 0.66) 0.658
B vs. D			-0.41 (-1.41, 0.59) 0.402
C vs. D			0.31 (-0.67, 1.29) 0.516
Age (per 10 years)	-0.37 (-0.81, 0.07) 0.098	-0.27 (-0.84, 0.29) 0.325	-0.36 (-0.81, 0.09) 0.108
Male vs. female	-0.13 (-0.71, 0.45) 0.648	-0.13 (-0.83, 0.57) 0.706	-0.16 (-0-81, 0.49) 0.610

Supplementary Table 3 (part 3 of 4). The estimates and the 95% confidence intervals of the association between each combination of six measures of glucose metabolism (A-F), as well as G: HbA1c (mmol/mol), H: log2(FPIR mU/L), and two measures of homeostasis model assessment (I) log2-transformed HOMA2-%B quantifying beta cell function, and (J) log2-transformed HOMA2-%S quantifying insulin sensitivity as outcomes and three measures of autoantibody status as predictors (count, status, combination group), adjusting for age and sex, estimated using linear models. The six outcome measures of glucose metabolism are: A: OGTT 2hr glucose (mmol/L), B. log2(OGTT glucose AUC), C: HbA1c (mmol/mol), D. log2(FPIR ml/L)), E: the median glucose (mmol/L) value based on a CGM from a 7-day sampling every 5 minutes, and F: The difference between the 75th and 25th percentiles of glucose (mmol/L) values based on a CGM from a 7-day sampling every 5 minutes. The outcomes were log2-tranformed as needed. The autoantibody information used as the main predictors was modeled as: the number of autoantibodies (possible values were 2, 3, or 4), autoantibody status for IAA, GADA, IA-2A and any of ZnT8(W/Q/R)A, with negative status being the reference, and the autoantibody combination group A-D (see Table 2) with group D as the reference. (Panel H in blue was reported in the main manuscript as well, it is included here for completeness).

**Outcome G: HbA1c (mmol/mol)**			
Covariates (predictors)	Model 1G (n = 57)<br>Est (95% CI) p-value	Model 2G (n = 57)<br>Est (95% CI) p-value	Model 3G (n = 57)<br>Est (95% CI) p-value
Number of autoantibodies	-0.09 (-1.42, 1.25) 0.899		
IAA (positive vs. negative)		-0.67 (-2.76, 1.43) 0.525	
GADA (positive vs. negative)		-1.35 (-5.20, 2.50) 0.484	
IA-2A (positive vs. negative)		0.14 (-2.08, 2.35) 0.901	
ZnT8(W/Q/R)A (positive vs. negative)		0.68 (-1.73, 3.10) 0.572	
Autoantibody combination group:			
A vs. D			0.36 (-2.39, 3.10) 0.796
B vs. D			0.09 (-2.80, 2.99) 0.949
C vs. D			1.70 (-1.21, 4.62) 0.246
Age (per 10 years)	0.55 (-0.40, 1.49) 0.249	0.66 (-0.33, 1.65) 0.188	0.66 (-0.30, 1.62) 0.171
Male vs. female	0.26 (-1.83, 2.36) 0.802	0.24 (-1.94, 2.42) 0.825	0.40 (-1.76, 2.55) 0.713

**Outcome H: log2(FPIR mU/L)**			
Covariates (predictors)	Model 1H (n = 52)<br>Est (95% CI) p-value	Model 2H (n = 52)<br>Est (95% CI) p-value	Model 3H (n = 52)<br>Est (95% CI) p-value
Number of autoantibodies	-0.39 (-0.83, 0.05) 0.084		
IAA (positive vs. negative)		-0.20 (-0.89, 0.49) 0.564	
GADA (positive vs. negative)		0.32 (-1.02, 1.65) 0.637	
IA-2A (positive vs. negative)		-0.40 (-1.12, 0.31) 0.262	
ZnT8(W/Q/R)A (positive vs. negative)		**-0.80 (-1.58, -0.02) 0.046**	
Autoantibody combination group:			
A vs. D			-0.45 (-1.39, 0.49) 0.345
B vs. D			-0.68 (-1.64, 0.27) 0.158
C vs. D			-0.17 (-1.18, 0.84) 0.736
Age (per 10 years)	0.15 (-0.16, 0.45) 0.343	0.09 (-0.23, .040) 0.580	0.17 (-0.16, 0.49) 0.307
Male vs. female	-0.14 (-0.83, 0.56) 0.695	-0.08 (-0.82, 0.65) 0.818	-0.24 (-0.98, 0.51) 0.525

Supplementary Table 3 (part 4 of 4). The estimates and the 95% confidence intervals of the association between each combination of six measures of glucose metabolism (A-F), as well as G: HbA1c (mmol/mol), H: log2(FPIR mU/L), and two measures of homeostasis model assessment (I) log2-transformed HOMA2-%B quantifying beta cell function, and (J) log2-transformed HOMA2-%S quantifying insulin sensitivity as outcomes and three measures of autoantibody status as predictors (count, status, combination group), adjusting for age and sex, estimated using linear models. The six outcome measures of glucose metabolism are: A: OGTT 2hr glucose (mmol/L), B. log2(OGTT glucose AUC), C: HbA1c (mmol/mol), D. log2(FPIR ml/L)), E: the median glucose (mmol/L) value based on a CGM from a 7-day sampling every 5 minutes, and F: The difference between the 75th and 25th percentiles of glucose (mmol/L) values based on a CGM from a 7-day sampling every 5 minutes. The outcomes were log2-tranformed as needed. The autoantibody information used as the main predictors was modeled as: the number of autoantibodies (possible values were 2, 3, or 4), autoantibody status for IAA, GADA, IA-2A and any of ZnT8(W/Q/R)A, with negative status being the reference, and the autoantibody combination group A-D (see Table 2) with group D as the reference. (Panel H in blue was reported in the main manuscript as well, it is included here for completeness).

**Outcome I: log2(HOMA2 %B)**			
Covariates (predictors)	Model 1I (n = 56)<br>Est (95% CI) p-value	Model 2I (n = 56)<br>Est (95% CI) p-value	Model 3I (n = 56)<br>Est (95% CI) p-value
Number of autoantibodies	-0.06 (-0.26, 0.14), 0.552		
IAA (positive vs. negative)		-0.13 (-0.45, 0.19), 0.409	
GADA (positive vs. negative)		-0.41 (-0.99, 0.17), 0.161	
IA-2A (positive vs. negative)		0.07 (-0.27, 0.41), 0.681	
ZnT8(W/Q/R)A (positive vs. negative)		-0.09 (-0.45, 0.28), 0.637	
Autoantibody combination group:			
A vs. D			-0.1 (-0.52, 0.33), 0.655
B vs. D			-0.14 (-0.59, 0.32), 0.548
C vs. D			0.02 (-0.43, 0.48), 0.926
Age (per 10 years)	0.01 (-0.13, 0.16), 0.866	0.02 (-0.13, 0.17), 0.78	0.02 (-0.14, 0.17), 0.831
Male vs. female	-0.23 (-0.55, 0.09), 0.160	-0.26 (-0.59, 0.07), 0.123	-0.24 (-0.58, 0.1), 0.159

**Outcome J: log2(HOMA2 %S)**			
Covariates (predictors)	Model 1J (n = 56)<br>Est (95% CI) p-value	Model 2J (n = 56)<br>Est (95% CI) p-value	Model 3J (n = 56)<br>Est (95% CI) p-value
Number of autoantibodies	-0.03 (-0.3, 0.24), 0.841		
IAA (positive vs. negative)		0.01 (-0.42, 0.45), 0.945	
GADA (positive vs. negative)		0.12 (-0.68, 0.91), 0.769	
IA-2A (positive vs. negative)		-0.14 (-0.61, 0.32), 0.541	
ZnT8(W/Q/R)A (positive vs. negative)		0.05 (-0.44, 0.55), 0.827	
Autoantibody combination group:			
A vs. D			-0.21 (-0.78, 0.36), 0.46
B vs. D			0.16 (-0.44, 0.76), 0.591
C vs. D			-0.21 (-0.81, 0.4), 0.497
Age (per 10 years)	-0.11 (-0.3, 0.09), 0.276	-0.10 (-0.31, 0.10), 0.319	-0.13 (-0.33, 0.07), 0.209
Male vs. female	0.2 (-0.24, 0.63), 0.364	0.22 (-0.24, 0.67), 0.346	0.21 (-0.24, 0.66), 0.353

Supplementary Table 4. The estimates, 95% confidence intervals and p-values of the association between two summary statistics of glucose (mmol/L) from a continuous glucose monitor (CGM) worn for 7 days (A: the median glucose (mmol/L) value based on a CGM from a 7-day sampling every 5 minutes (Supplementary Figure 1), and B: The difference between the 75th and 25th percentiles of glucose (mmol/L) values based on a CGM from a 7-day sampling every 5 minutes) (Supplementary Figure 1), and glucose measures based on OGTT (2h glucose, area under the glucose curve), IvGTT (first phase insulin response (FPIR)), as well as HbA1c. Measures were log2-tranformed as needed. All models were adjusted for age and sex. For models 1A-4A (top panel) the outcome was the median, for Models 1B-4B (bottom panel) the outcome was the range of IQR. This analysis was based on n = 23 participants for model 1A/B, and for n = 24 for all other models. See supplementary Figure 1 below for a visualization of the two outcomes based on the CGM data.

Outcome A	Covariates (predictors)	Model 1A (n = 23) Est (95% CI)	Model 2A (n = 24) Est (95% CI)
Median glucose (mmol/L) value based on a CGM from a week-long sampling every 5 minutes	log2(FPIR)	**-0.30 (-0.54, -0.06) 0.016**	
	HbA1c (mmol/mol)		**0.11 (0.04, 0.17) 0.002**
	OGTT 2h Glucose		
	log2(OGTT Glucose AUC)		
	Age (per 10 years)	-0.01 (-0.44, 0.42) 0.951	0.05 (-0.32, 0.43) 0.777
	Male vs. female	-0.27 (-0.83, 0.28) 0.318	-0.12 (-0.60, 0.36) 0.606
	Covariates (predictors)	Model 3A (n = 24) Est (95% CI)	Model 4A (n = 24) Est (95% CI)
	log2(FPIR)		
	HbA1c (mmol/mol)		
	OGTT 2h Glucose	**0.12 (0.05, 0.20) 0.002**	
	log2(OGTT Glucose AUC)		**0.85 (0.19, 1.51) 0.014**
	Age (per 10 years)	-0.11 (-0.46, 0.25) 0.530	-0.21 (-0.60, 0.18) 0.277
	Male vs. female	-0.02 (-0.51, 0.48) 0.948	-0.17 (-0.69, 0.36) 0.518

Outcome B	Covariates (predictors)	Model 1B (n = 23) Est (95% CI)	Model 2B (n = 24) Est (95% CI)
Range of the IQR of glucose (mmol/L) values based on a CGM from a week-long sampling every 5 minutes	log2(FPIR)	**-0.32 (-0.54, -0.10) 0.007**	
	HbA1c (mmol/mol)		**0.09 (0.02, 0.15) 0.014**
	OGTT 2h Glucose		
	log2(OGTT Glucose AUC)		
	Age (per 10 years)	-0.08 (-0.47, 0.32) 0.693	-0.12 (-0.53, 0.29) 0.552
	Male vs. female	-0.03 (-0.54, 0.49) 0.910	-0.02 (-0.55, 0.50) 0.927
	Covariates (predictors)	Model 3B (n = 24) Est (95% CI)	Model 4B (n = 24) Est (95% CI)
	log2(FPIR)		
	HbA1c (mmol/mol)		
	OGTT 2h Glucose	**0.11 (0.04, 0.19) 0.005**	
	log2(OGTT Glucose AUC)		**0.98 (0.37, 1.59) 0.003**
	Age (per 10 years)	-0.24 (-0.61, 0.13) 0.185	-0.34 (-0.70, 0.02) 0.063
	Male vs. female	0.09 (-0.43, 0.60) 0.725	-0.02 (-0.51, 0.46) 0.920

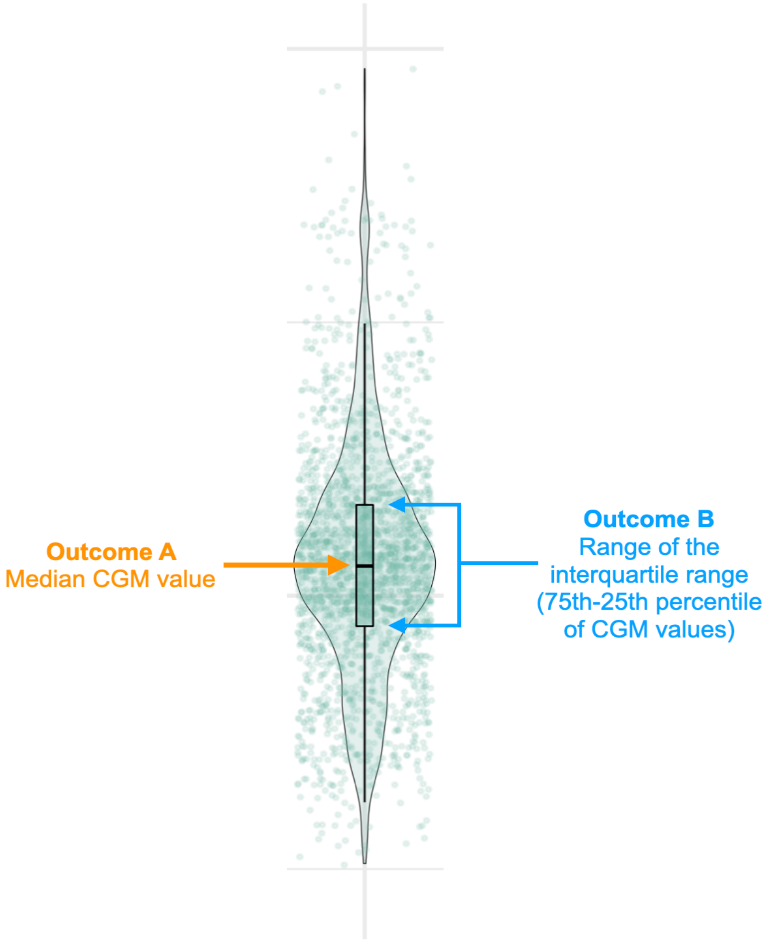

Supplementary Figure 1. Two summary statistics of glucose (mmol/L) from a continuous glucose monitor (CGM) worn for 7 days: the median glucose (mmol/L) value based on a CGM from a 7-day sampling every 5 minutes (A) and the difference between the 75th and 25th percentiles of glucose (mmol/L) values based on a CGM from a 7-day sampling every 5 minutes (B).
